# Classification of the Use of Online Health Information Channels and Variation in Motivations for Channel Selection: Cross-sectional Survey

**DOI:** 10.2196/24945

**Published:** 2021-03-09

**Authors:** Di Zhang, Zhen Shi, Hongchao Hu, Gang (Kevin) Han

**Affiliations:** 1 The Research Center of Journalism and Social Development Renmin University of China Beijing China; 2 Greenlee School Journalism and Communication Iowa State University Ames, IA United States

**Keywords:** search, browse, scan, health information seeking, channel selection, health information, health education, health communication, online media

## Abstract

**Background:**

Existing health education and communication research routinely measures online channel use as a whole by, for example, evaluating how frequently people use the internet to search for health information. This approach fails to capture the complexity and diversity of online channel use in health information seeking. The measurement of generic online channel use may cause too much error, and it lends no support to media planning in public health promotion campaigns or scholarly research involving online channel use.

**Objective:**

This study intends to present a thorough picture of patterns of online health information channel use and classify the use of various types of online health information channels, including WeChat, microblogs, web portals, search engines, mobile apps, and online forums. Under the framework of the risk information seeking and processing model, this study also analyzes the differences in individuals’ motivations for channel selection to offer further evidence to validate the classification scheme.

**Methods:**

This study sampled 542 Chinese internet users in Beijing. The average age of the respondents was 33 years, female respondents accounted for 52.0% (282/542) of the sample, and the average monthly income ranged from US $900 to $1200. The study surveyed the use of 13 commonly used online health information channels and various sociopsychological factors associated with online health information seeking.

**Results:**

This study derived 3 categories of online health information channels: searching, browsing, and scanning channels. It was found that the use of online searching channels was affect driven ﻿(B=0.11; β=0.10; *P*=.02) and characterized by a stronger need for health knowledge ﻿(B=0.09; β=0.01; *P*<.001). The use of browsing channels was directly influenced by informational subjective norms ﻿(B=0.33; β=0.15; *P*=.004) and perceived current knowledge ﻿(B=0.007; β=0.09; *P*=.003). The use of scanning channels was mainly influenced by informational subjective norms ﻿(B=0.29; β=0.15; *P*=.007).

**Conclusions:**

The results of this study suggest that health communication practitioners and scholars may consider measuring the use of internet, new media, or online media more precisely instead of simply asking the public about the frequency of online channel use or internet use in the acquisition of health information. Scholars and practitioners may consider measuring the use of online health information channels by using the 3-category scheme described in this study. Future research is encouraged to further explore how people process health information when using different online channels.

## Introduction

### Background

People across the globe increasingly name online channels as one of their top choices when acquiring health information and knowledge [1], which accordingly attracts scholarly attention. Most existing studies treat online channels as a whole by, for example, evaluating how frequently people use the internet to search for health information [2,3]. In this study, online health information channels refer to online communication media and applications that collect health information and knowledge from sources, repackage them, and then distribute them to people [4]. However, terms such as “new media,” “online media,” and “internet” are very generic. Treating the internet as a whole does not reflect the complexity and diversity in the use of online health information channels [5]. Therefore, the first goal of this study is to survey the use of various types of online health information channels, thus presenting a more nuanced picture of the use of online health information channels.

In addition to describing the channel use frequencies, the second goal is to classify the use of various online health information channels into different categories from the perspective of information-seeking behaviors, which differs from the existing division method. When there is a need to analyze online channels separately, health communication researchers routinely divide online health information channels into news portals (such as news websites and health websites) and social media, as in the case of the Health Information National Trends Survey. This classification scheme is based on the differences in the diversity of content creators, platform structure, and connections between users [6], which is inherently the perspective of the platform designers and operators rather than the users. However, this perspective is increasingly incompatible with consumer-centered health communication campaign design, which assumes that satisfying individual needs is the key to effective campaigns [7]. In the era of the internet, it is more noticeable that individuals select communication channels to meet their felt needs [8]. Given that individual needs drive different styles of information-seeking behaviors, such as searching, browsing, and scanning [9,10], the development of an information-seeking, behavior-based channel classification scheme can be potentially more helpful for contemporary health communication researchers and practitioners, who would be able to use fewer measurement items while increasing the validity of the measurement in both academic and formative research of health information channel use. The development of such a scheme is also feasible because different channel types can fulfill different information-seeking strategies [11].

Third, this study examines the factors associated with online health information channel selection under the framework of the risk information seeking and processing (RISP) model, which depicts the various sociopsychological factors behind information seeking and processing [12]. The results of the study can contribute to the literature on RISP by explaining the variance in channel selection, which is a crucial part of information seeking that is underexplored [13].

### Literature Review

#### Classification of Online Channel Use and Health Information–Seeking Behaviors

With the rise of the internet and mobile phones, people in countries like the United States and China frequently search for health information on the internet [14,15]. Additionally, evidence collected in multiple countries revealed that people who search for health information use various types of online channels, such as search engines, health web portals, social networking sites, and online support groups [14,16,17].

This study intended to pinpoint the underlying patterns by classifying the use of online health information channels into clusters based on health information acquisition behaviors, which differs from the existing division method from the perspective of platform designers and operators. Current research on health information seeking suggests 3 types of behaviors: searching, browsing, and scanning [9,10]. People acquire health information mainly through 2 routes, active seeking and scanning [9]. Active seeking refers to an intentional process of acquiring health information, which implies more active efforts in health information seeking [9,18]. Research in library and information science and health informatics implies that active seeking can be further divided into searching and browsing, which vary in the degree of specificity of information seeking [19]. In health information, searching, which is directed, refers to users searching the internet for answers related to specific diseases or symptoms [10,20]. Browsing, which is undirected and motivated by curiosity, refers to users browsing health information without the intent to acquire knowledge about specific diseases or symptoms, consuming health information regularly and habitually, and following the structure and layout of information prepared by the publisher of the website or account [10,20]. In contrast, scanning is defined as a process in which people both encounter health information or knowledge while engaged in tasks unrelated to health and make a decision to process it [9], which is an effort less active than active seeking [21].

Different channel types can fulfill different information-seeking strategies because of the interface designs and functionalities of these channels [11]. According to interviews before data collection, people generally use a specific online channel to engage in the primary information acquisition activity. However, one particular channel can be suitable for more than one type of health information acquisition behavior. For instance, web portals can apply to both browsing and scanning, so web portals fall into both categories in this study. From what has been discussed above, the following research question was proposed: What are the patterns of online health information channel use?

#### Health Information Channel Selection and RISP

As opposed to many existing channel choice studies that emphasize the influences of channel characteristics (eg, ease of use, interactivity, privacy, and media scale) [22,23], this study explains the channel choice using an audience-centered approach because channel use depends on audience needs, particularly their psychological needs [4,24,25]. Specifically, the study used RISP as the theoretical model, which combines theories such as the heuristic-systematic model and the theory of planned behavior [12,26-29]. RISP was chosen for two other reasons. First, RISP has often been applied in the context of health risks, such as environmental health [30], vaccines [31], and clinical trial enrollment [32]. Second, channel choice can reflect different combinations of information-seeking and processing strategies, two major dependent variables in RISP. Different types of online health information channel use vary in information-seeking strategies (routine [nonactive] vs nonroutine [active]) [11,33] and may result in differences in information processing (heuristic vs systematic) [12]. In RISP, a combination of the 4 attributes in information seeking and processing results in a fourfold typology: (1) routine (ie, habitual, ritual) seeking with heuristic processing, (2) routine seeking with systematic processing, (3) nonroutine seeking with heuristic processing, and (4) nonroutine seeking with systematic processing [12]. In reference to the abovementioned conceptual definitions of browsing, searching, and scanning, channel choice based on information-seeking strategies may represent different combinations of information-seeking and processing strategies, thus making RISP an ideal theoretical framework. For instance, searching (active but directed) is similar to nonroutine seeking with systematic processing.

**Figure 1:**
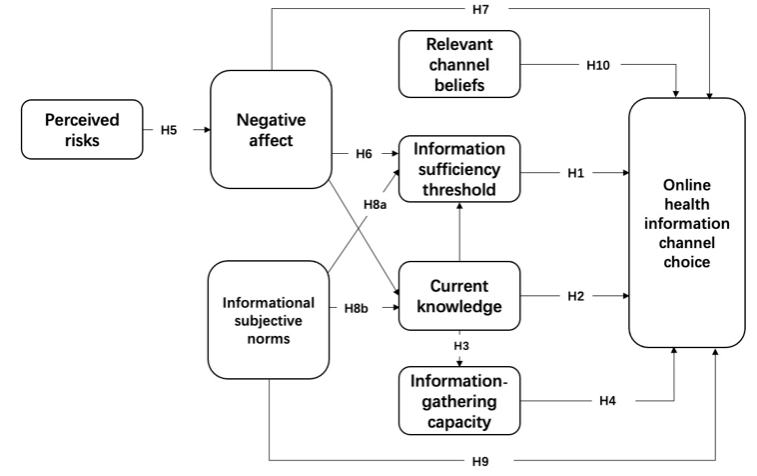
Conceptual model. The lines between current knowledge and negative affect and information sufficiency threshold were added for the purpose of statistical control. Thus, these lines are unmarked. H: hypothesis.

RISP studies primarily focus on the following key predictor variables: information insufficiency, informational subjective norms, perceived hazard characteristics, affective responses, relevant channel beliefs, and perceived information-gathering capacities. Figure 1 shows the conceptual model consisting of predictor and outcome variables.

Information insufficiency refers to the perceived cognitive need for additional information, which is the perception of a gap between one’s existing knowledge and a level of knowledge sufficient to handle risks confidently (information sufficiency threshold) [12,33]. Previous studies confirm that information insufficiency leads to active information seeking and systematic processing [34,35]. Additionally, perceived knowledge is essentially the complement to the sufficiency threshold link for purposes of assessing regressed change and difference scores; RISP meta-analyses and marketing research suggest that current knowledge accounts for a substantial amount of variance in information seeking [36] and systematic processing [37-39]. Since previous RISP studies did not directly examine channel choice, 2 sets of hypotheses without directions were proposed for the 2 variables:

- H1: Controlling for current knowledge, the information sufficiency threshold is related to the use of searching sites (H1a), browsing sites (H1b), and scanning sites (H1c).
- H2: Current knowledge is related to the use of searching sites (H2a), browsing sites (H2b), and scanning sites (H2c).

Perceived information-gathering capacity refers to one’s perceived abilities to acquire and process risk-related information [26,29,33], which is positively associated with current knowledge and information seeking [30,40]. Motivated by information insufficiency or pressure from social norms to seek information, people may need confidence that they are able to perform information-seeking tasks [41]. Thus, the following were proposed:

- H3: Current knowledge is positively associated with perceived information-gathering capacity.
- H4: Perceived information-gathering capacity is related to the use of searching sites (H4a), browsing sites (H4b), and scanning sites (H4c).

Perceived hazard characteristics are one’s cognitive evaluation of the nature of hazards [33]. Perceived hazard characteristics include personal control, trust in risk management, perceived threats to personal values, and risk judgment [12,33]. However, this study focused on the perceived probability of contracting a disease (short for perceived risk afterwards) because most people are unlikely to have serious diseases. Previous RISP studies have found that risk judgment can potentially increase the level of negative affect [12,41,42], which further contributes to more information seeking and processing via one’s information insufficiency level or directly [40]. Thus, the following was proposed:

- H5: Perceived risks are positively associated with negative affect.

Affective responses are induced by risk perception and can lead to a higher level of information insufficiency [12]. Negative affect was found to influence information-seeking behaviors directly [40]. Thus, the following were proposed:

- H6: Negative affect is positively associated with the information sufficiency threshold.
- H7: Negative affect is related to the use of searching sites (H7a), browsing sites (H7b), and scanning sites (H7c).

Informational subjective norms are the perceived socioenvironmental influence on an individual’s subjective assessment of information held to handle a given risk and motivation to seek and process information [33]. Informational subjective norms consist of two dimensions, injunctive and descriptive [35], which have been found to influence information seeking [36,43]. Thus, the following were proposed:

- H8: Informational subjective norms are positively associated with the information sufficiency threshold (H8a) and current knowledge (H8b).
- H9: Informational subjective norms are related to the use of searching sites (H9a), browsing sites (H9b), and scanning sites (H9c).

Relevant channel beliefs refer to people’s beliefs about channels that carry risk-related information, including their trustworthiness and usefulness, that could influence information seeking and processing either as a main effect or as a moderator [12,33]. Thus, the following was proposed:

- H10: Relevant channel beliefs are related to the use of searching sites (H10a), browsing sites (H10b), and scanning sites (H10c).

## Methods

### Sample

This study used survey data collected in 2015 because, according to the surveys by the China Internet Network Information Center in 2015 and 2020, penetration rates of major new media applications related to this study (search engines, news applications, and mobile communication apps, including WeChat and microblogs) did not change much between 2015 and 2020. The survey targeted residents in Beijing because Beijing had China’s highest internet penetration rate in 2015, at 76.5% [44], which partially excludes the problem of the physical digital divide. Given its vibrant internet activities and high economic level (including gross domestic product per capita), which is comparable to developed countries, the choice of Beijing residents as the sample renders the results useful for scholars in Western countries such as the United States, where most health communication studies take place.

The researchers hired Sojump, a Chinese online panel company, to collect the data because this study only focused on internet users, which allowed the researchers to remove those without internet access and thus the problem of the digital divide. This study used quota sampling to draw participants, which is common for online panel surveys [45]. The quota sampling scheme used age group and gender as the criteria in designing subgroups (an equal number of respondents in the 8 subgroups). Age in this study was divided into the categories of 18 to 25 years, 26 to 30 years, 31 to 40 years, and 41 years and older. The researchers oversampled people older than 30 years because that age is positively related to perception of health risks, which may elicit health information seeking. In the panel, 170,000 participants were from Beijing. For this study, Sojump randomly sent survey invitations in September 2015 to 9500 people within the panel who were registered and confirmed as from Beijing. The data collection process lasted for 10 days and resulted in a sample size of 542. The average age of the respondents was 33 years old, female respondents accounted for 52.0% (282/542) of the sample, and the average monthly income ranged from US $900 to $1200.

### Dependent Variables

The outcome variable was the extent to which an individual uses a particular online channel to obtain health information, and this was measured on a 7-point scale. According to preliminary in-depth interviews with Chinese health communication practitioners and users, the researchers pinpointed 9 types of commonly used online health information channels. Given that one channel can be suitable for more than one type of information acquisition behavior, the researchers measured the frequencies of 13 types of online health information channel use (Table 1).

**Table 1:** Descriptive statistics of online channel use in Beijing, China (N=542).

Category and online health information channels		Mean^a^ (SD)
**Browsing channels^b^**		
	WeChat^c^ official accounts (ie, health-related official accounts, similar to Facebook pages)	4.58 (1.92)
	Microblogs (ie, health microblogs, similar to Twitter)	4.22 (2.05)
	Web portals (ie, health section, similar to Yahoo)	3.85 (1.91)
	Online professional health sites (similar to WebMD)	3.72 (1.98)
	Mobile phone health apps	3.67 (2.05)
	Online forums (ie, forums related to a particular disease or health problem)	3.61 (2.02)
**Searching channels^b^**		
	Search engines	5.29 (1.64)
	Online encyclopedia sites	4.61 (1.84)
	Question-and-answer sites	4.51 (1.85)
**Scanning channels^d^**		
	WeChat Moments (similar to Facebook News Feed)	5.12 (1.66)
	Web portals (ie, nonhealth sections)	4.52 (1.67)
	Microblogs (ie, nonhealth microblogs)	4.38 (1.81)
	Online forums (ie, nonhealth forums)	4.06 (1.82)

^a^Scale of 1 to 7: 1=never, 7=very often.

^b^Browsing channels and searching channels are the channels for active seeking.

^c^WeChat is China’s largest mobile messenger service and its functionality is similar to Facebook.

^d^Scanning channels are the channels for incidental exposure, passive exposure, and routine seeking.

When wording the question items, the researchers took into account whether a channel was for active seeking or incidental exposure (scanning). For active-seeking channels, the survey participant was asked, “Over the past year, to what extent have you used the following channel to acquire information or knowledge related to generic health, disease prevention/treatment and healthy living?” [9]. For scanning channels, the survey participant was asked “Over the past year, to what extent did you accidently pay attention to information or knowledge related to generic health, disease prevention/treatment and healthy living while engaged in media tasks other than health information search?” [9]. As shown in Table 1, search engines (active) and WeChat Moments (incidental exposure, similar to Facebook News Feed) were the 2 most frequently used channels (search engines: mean 5.29, SD 1.64; WeChat Moments: mean 5.12, SD 1.66). Online health forums and mobile health apps were the 2 least used channels (health forums: mean 3.61, SD 2.02; health apps: mean 3.67, SD 2.05).

### Predictor Latent Variables

This study measured 7 RISP predictors. Table 2 lists specific survey question items and their descriptive statistics.

**Table 2:** Descriptive statistics of RISP predictor variables (N=542).

Variables and questions			Mean (SD)
**Information insufficiency^a^** **(1-100 scale)**			
	Perceived current health knowledge: Estimate your knowledge of health, with 1=knowing nothing and 100=knowing everything you could possibly know about health maintenance.	59.88 (17.13)	
	Sufficiency threshold: This time, using that same scale, estimate how much knowledge you think you need on health maintenance.	73.27 (20.56)	
**Information subjective norms (1-5 scale)^b^**			
	Injunctive: My family expects me to seek health knowledge.	3.78 (0.88)	
	Injunctive: My friends expect me to seek health knowledge.	3.62 (0.94)	
	Descriptive: People in my life whose opinions I value seek health knowledge.	3.53 (0.86)	
**Perceived risk** **(1-5 scale)^c^**			
	My health may face problems in the next year.	2.51 (1.09)	
	In the next year, I may possibly suffer from diseases that may impact my job or life.	2.44 (1.18)	
	In the next year, I am confident about my health (reverse coding).	2.20 (0.97)	
**Negative affect (1-5 scale)^d^**			
	How much of the following do you feel about your health? Not worried…Very worried	3.11 (1.05)	
	How much of the following do you feel about your health? Not anxious…Very anxious	2.71 (1.14)	
**Relevant channel beliefs (1-5 scale)^e^**			
	To what extent do you trust health information on web portals?	3.53 (0.74)	
	To what extent do you trust health information on social media?	3.46 (0.82)	
	To what extent do you trust health information on mobile phone apps?	3.45 (0.92)	
**Perceived information-gathering capacities (1-5 scale)^f^**			
	It is difficult to find health knowledge (reverse coding).	3.39 (1.06)	
	I don’t know where to find health knowledge (reverse coding).	3.37 (1.07)	
	I have a hard time understanding health knowledge (reverse coding).	3.38 (1.23)	
Age			33.01 (9.19)
Gender (female), %			52.0 (—)
Income			3.76 (1.51)

^a^In the questionnaire, the item “current knowledge” was presented first, followed by the item “sufficiency threshold.”

^b^α=.70.

^c^α=.81.

^d^α=.79.

^e^α=.61.

^f^α=.82.

Information insufficiency was assessed using 2 items: perceived current health knowledge and information sufficiency threshold [12]. In the analysis, information insufficiency’s influence on health information seeking was evaluated by modeling the information sufficiency threshold when controlling for the self-assessment of current health knowledge.

Informational subjective norms included injunctive and descriptive norms [35]. The former refers to important others’ attitudes toward one’s behaviors, while the latter refers to important others’ behaviors. A total of 3 items were used.

Perceived risks evaluated one’s self-assessed probability of suffering from diseases or encountering health problems, which is consistent with previous RISP research [36]. This construct consisted of 3 items with a 5-point scale.

Negative affect assessed one’s negative feeling related to their health status [36]. It consisted of 2 items.

Relevant channel beliefs evaluated one’s belief that online channels are capable of supplying trustworthy information [35]. In this study, the 3 items specifically tapped into the dimension of trust, since Chinese online media are often criticized for containing misinformation related to health.

Perceived information-gathering capacities assessed one’s belief that they are capable of accessing and understanding health information [35]. Three 5-point items were used to measure this concept.

## Results

### Overview

The researchers analyzed the data using structural equation modeling techniques. Initially, the researchers reduced the use of 13 online channels into 3 groups of variables, which were used as endogenous variables [32]. Then, the structural model was built. The lavaan package in R was used to test hypotheses.

### Measurement Model

Three measurement models were constructed and compared (Multimedia Appendix 1 illustrates the specific measurement model–building procedures). The third measurement model had the best model fit (Table 3). The third measurement model classified the 13 channel use variables into 3 groups: browsing, searching ,and scanning channels. Additionally, the third measurement model correlated the error terms of 6 pairs of variables of channel use that have moderate to high levels of correlation due to shared technical platforms (Multimedia Appendix 2). For instance, people who prefer health sections of web portals are likely to have the habit of using web portals, which raises their chances of encountering health messages when using web portals for tasks unrelated to health.

**Table 3:** Model fit statistics.^a^

Model		Chi-square (*df*)		χ^2^/*df*		RMSEA^b^		CFI^c^		SRMR^d^	
**Measurement model**											
	Model 1: Baseline (2-factor)^e^		1149.1 (303)		3.79		0.072		0.86		0.059
	Model 2. Revised (3-factor)^f^		904.8 (296)		3.06		0.062		0.90		0.052
	Model 3: Revised + correlated error		638.0 (290)		2.20		0.047		0.94		0.049
**Structural model**											
	Model 1: Baseline conceptual		923.0 (343)		2.69		0.056		0.91		0.081
	Model 2: Revised		931.7 (351)		2.65		0.055		0.91		0.083

^a^Recommended cutoff points for model fit indices [46-48]: SRMR of <0.08; RMSEA of <0.08; CFI of >0.90 (ideally CFI of ≥0.95); χ^2^/*df* of <3. Hu and Bentler [47] suggest a 2-index presentation strategy, recommending a RMSEA of 0.06 or lower and a SRMR of 0.09 or lower.

^b^RMSEA: root mean square error of approximation.

^c^CFI: comparative fit index.

^d^SRMR: standardized root mean residual.

^e^2-factor: active seeking and scanning channels.

^f^3-factor: searching, browsing, and scanning channels.

The results from the measurement model suggested that the use of online health information channels can be divided into 3 categories: browsing, searching, and scanning channels (Table 1). Browsing channels consist of health sections of web portals (B=1.00; β=0.72; *P*<.001), professional health sites (B=1.01; β=0.70; *P*<.001), microblogs (B=1.01; β=0.67; *P*<.001), WeChat official accounts (B=0.91; β=0.64; *P*<.001), mobile health apps (B=1.08; β=0.73; *P*<.001), and online health forums (B=1.09; β=0.75; *P*<.001).

Searching channels include search engines (B=1.00; β=0.65; *P*<.001), online encyclopedia sites (B=1.35; β=0.79; *P*<.001), and question-and-answer sites (B=1.35; β=0.78; *P*<.001).

Scanning channels encompass incidental exposure to health information on web portals (B=1.00; β=0.73; *P*<.001), microblogs (B=1.02; β=0.69; *P*<.001), WeChat Moments (B=0.68; β=0.50; *P*<.001), and online forums (B=1.14; β=0.76; *P*<.001)**.**

### Structural Model

**Figure 2:**
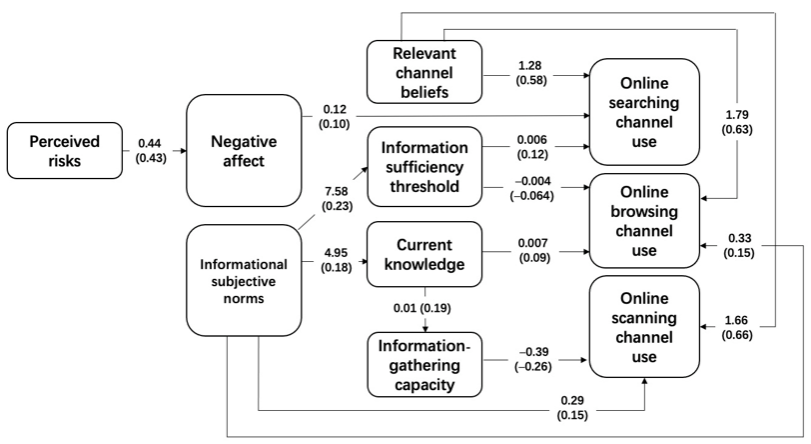
Revised model. The numbers in parentheses are standardized coefficients and the numbers preceding the parentheses are unstandardized coefficients.

The bottom of Table 3 presents the model fit indices for the structural model. Two structural models, conceptual and revised (removing the nonsignificant paths), were compared. The popular model fit indices were roughly the same. The revised model (Figure 2) was retained, since the likelihood test suggested that the 2 competing models were not significantly different (Δχ^2^_1_=8.7, P=.37). Figure 2 presents test results of H1 to H10.

H1 examined the extent to which the information sufficiency threshold was associated with online channel choice. According to the results, the information sufficiency threshold was positively associated only with the use of the online searching channel (B=0.09; β=0.01; *P*<.001) and was negatively related to the use of online browsing channels (B=–0.004; β=–0.064; P=.03). H1a and H1b were supported. However, the information sufficiency threshold was not related to the use of scanning channels, and H1c was not supported. H2 analyzed in what way current knowledge was related to online channel selection. It was found that current knowledge was only positively associated with the use of online browsing channels (B=0.007; β=0.09; P=.003), not searching (H2a) and scanning (H2c) channels. Thus, only H2b was supported.

H3 examined the positive association between current knowledge level and perceived information-gathering capacities and was supported (B=0.01; β=0.19; *P*<.001). H4 examined the relationship between perceived information-gathering capacities and online channel selection. It was found that perceived information-gathering capacities had negative associations with online scanning channel use (B=–0.39; β=–0.26; *P*<.001). Thus, H4b was supported. Conversely, perceived information was not related to the use of online searching and browsing channels, and H4a and H4b were not supported.

H5 predicted the positive association of perceived risks with negative affect, and it was supported (B=0.44; β=0.43; *P*<.001). H6 predicted that negative affect would be positively associated with the information sufficiency threshold. However, this was not supported. H7 examined the extent to which negative affect was associated with channel choice. The results suggested that negative affect was positively associated only with online searching channel use (B=0.11; β=0.10; *P*=.02) and not the use of the other 2 types of online channels. Thus, only H7a was supported.

H8a and H8b tested the positive relationship between informational subjective norms and the information sufficiency threshold and current knowledge, both of which were supported. The results suggested that informational subjective norms were positively associated with the information sufficiency threshold (B=7.58; β=0.23; *P*<.001) (H8a) and current knowledge (B=4.95; β=0.18; *P*<.001) (H8b). H9 examined the relationship between informational subjective norms and online channel selection. The results revealed that informational subjective norms were directly related to online browsing (B=0.33; β=0.15; *P*=.004) (H9b) and scanning channels (B=0.29; β=0.15; *P*=.007) (H9c). However, informational subjective norms did not have a direct association with the use of searching channels (H9a).

H10 tested whether relevant channel beliefs were related to online channel use, which was supported. Additionally, the sizes of the coefficients were roughly the same (for searching channels [H10a]: B=1.28; β=0.58; *P*<.001; for browsing channels [H10b]: B=1.79; β=0.63; *P*<.001; for scanning channels [H10c]: B=1.66; β=0.66; *P*<.001).

## Discussion

### Overview

This study described how people in Beijing, China, use online channels to acquire health information. The results suggested that the 13 commonly used online health information channels can be divided into 3 categories: browsing, searching, and scanning channels (Table 1). These were also corroborated by the underlying motivational mechanism.

### Diverse Use of Online Health Information Channels

This study found that Chinese people use online health information channels to different extents. Along with search engines, China’s largest messenger service, WeChat (WeChat Moments, a type of scanning channel), was reported as one of the 2 most frequently used channels. Other frequently used channels included WeChat official accounts (similar to Facebook pages), online encyclopedia sites, question-and-answer sites, microblogs (browsing and scanning), and web portals (scanning). The descriptive statistics clearly show that although search engines were the most frequently used, the importance of social media in online health information acquisition in China outweighs that of the channels run by institutions, which is in line with previous studies that found that people in East Asia prefer social media when seeking health information [17].

### Patterns of Online Health Information Channel Use

The confirmatory factor analysis further revealed that the use of online health information channels can be classified into 3 categories. From the perspective of the theory of channel complementarity [8], the variable clustering pattern may hint that people use different online channels with similar functions complementarily to obtain health information. Search engines, online encyclopedias, and question-and-answer sites are grouped together. When people use search engines, they use specific keywords to actively search for information related to specific health concerns. Online encyclopedia sites are user-generated reference “books” in which people also commonly use specific keywords to query information. Additionally, question-and-answer site users with specific questions in mind actively solicit answers from fellow users, which is similar in nature to user behaviors on search engines and online encyclopedias. Therefore, search engines, online encyclopedias, and question-and-answer sites are online searching channels that users mainly use to search for answers related to health concerns. An alternative explanation might be that, from researchers’ anecdotal observations, search engines in China often return top results linking users to online encyclopedia sites and question-and-answer sites, which suggests that these two types of sites successfully apply search engine optimization strategies.

Web portals (eg, health sections), official accounts on WeChat, microblogs (eg, health microblog accounts), professional health sites, mobile health apps, and online health forums are combined into a separate group: online browsing channels. The content and layout of the content on the first 5 online channels are commonly prepared by professional editors; users normally follow the structure of information prepared by the publisher [10]. It is also noted that online health forums join the other 5 channels mentioned earlier. While online health forums allow users to solicit answers about specific questions, many users are usually spectators scrolling through the posts and threads of others. Thus, the use of online health forums is more similar to the use of health sections of web portals than the use of search engines and question-and-answer sites.

Updates on WeChat Moments and microblogs, web portals (eg, general news section), and online forums not related to health are grouped together as online scanning channels. Users encounter health information on these channels when engaged in tasks other than active health information seeking.

### Differences in Sociopsychological Mechanisms for Channel Choice

This study further pinpointed the variation in motivation to use different types of health information channels from the perspective of RISP [12,33] and showed the validity of RISP in explaining information channel selection. The differences in sociopsychological mechanisms for channel selection corroborated the validity of the results generated by confirmatory factor analysis.

The use of searching channels, including search engines, question-and-answer sites, and online encyclopedia sites, is motivated by the intention to acquire more health knowledge (information sufficiency threshold) and by negative affect. Additionally, online searching channel use is indirectly caused by perceived risks via negative affect and indirectly influenced by informational subjective norms via the information sufficiency threshold. An individual who chooses search engines to search for specific disease-related keywords to reduce uncertainties is likely to feel threatened by health problems and have a stronger need for health knowledge.

The use of online scanning channels, including WeChat Moments, microblogs, online forums, and web portals, is driven primarily by informational subjective norms. An individual who pays attention to health information or knowledge on online scanning channels is unlikely to be concerned about their health status or increasing their knowledge. The use of online scanning channels to obtain health knowledge is attributed more to pressure from the individual’s interpersonal social network.

Similar to the use of online scanning channels, the use of online browsing channels is also directly driven by influence from one’s social network. Additionally, informational subjective norms predict the use of browsing channels via current knowledge. Browsing content on online professional health sites, for instance, does not necessarily mean that an individual can immediately benefit from the content read. Only if the individual understands the potential long-term benefits of that information or knowledge can they take the time to process the information. A higher level of current knowledge may help individuals be aware of the long-term benefits of health knowledge accruement. In other words, the use of browsing channels is comparable to reading lengthy books, while the use of searching channels is similar to looking up the definition of a term in a dictionary.

Interestingly, information-gathering capacity was negatively associated with the extent to which respondents used scanning channels. Being able to locate health knowledge might prevent users from using scanning channels because they might believe in other, more efficient routes of obtaining health information, such as those under the category of searching or browsing channels.

### Practical Implications

The results of the study suggest that health communication practitioners and scholars should measure “internet,” “new media,” and “online media” more precisely instead of simply asking the public about the frequency of internet use in health information acquisition. The measurement of generic internet use may cause too much error, and it lends no support to media planning in a public health promotion campaign.

More importantly, as contemporary health care consumers reside in a multichannel environment [49], health communication practitioners and scholars may consider developing more appropriate methods of classifying these channels to better manage them. As mentioned above, this study devises a new classification scheme based on health information–seeking behaviors. Practitioners may consider categorizing online channels using the new scheme generated by this study, which may better cater to the needs of individuals planning consumer-centered health communication campaigns.

The channel choice pattern and underlying sociopsychological mechanisms generate useful insights to improve health information strategies as well. We found that the use of online searching channels was uniquely driven by the need for more health knowledge and by stronger negative affect. In other words, people who use search engines, online encyclopedias, and question-and-answer sites may readily accept the answers found through these channels and use the knowledge to guide their health behaviors if they find the answers plausible. However, these 3 types of channels are often dominated by nonmedical professionals and possibly contain misinformation [50], so professional health agencies may consider establishing closer partnerships with these channels.

It was found that the use of online browsing channels was uniquely driven by self-reported current knowledge level, which implies that people with higher health literacy are more likely to choose browsing channels. Since most browsing channels are run by institutions, these channels and websites should focus on the provision of more advanced health knowledge. The basics should be left to sites such as online encyclopedias.

This study also found that the likelihood of using online scanning channels was primarily influenced by informational subjective norms. Additionally, health culture, which makes health a shared value, is beneficial to people’s health behaviors [51]. This means that if we could build a culture of health conducive to the habitual acquisition of health knowledge, users would be likely to process at least some health information encountered on online scanning channels. However, it should be noted that scanning channels may also contain a considerable amount of health content generated by nonprofessionals. Accuracy of online health information is always a concern [50], so practitioners should involve themselves as much as possible in such channels.

### Limitations and Future Studies

This study is not without limitations. Constrained by budgets, this study used an online panel to collect data. Although the sample covered a range of age groups, this study did not sample enough older adults, which to some extent limited the generalization of the results to the entire population. Additionally, online panels consist of respondents who are paid to respond to the survey questions. These respondents may differ from the general population, which may bias the results of the study.

Since this study revealed that motivations behind using different types of channels differ, future studies may further explore the differences in the impacts of online channel use on health knowledge gains and behavioral changes. Moreover, future research is advised to further explore how people process information when using different types of online health information channels. The fourfold typology of information seeking and processing suggests that different information-seeking strategies may possibly entail different styles of information processing. Researchers do not examine online health information channel use and selection for the sake of merely understanding channel selection; instead, the ultimate goal of such research is to have a better understanding of how different channel choices influence changes in people’s cognition, attitudes, and behaviors.

## Appendix

Multimedia Appendix 1 Model building.

Multimedia Appendix 2 Correlation tables.

## Abbreviations

RISP: risk information seeking and processing
