# Supplementary material for: Classification of the Use of Online Health Information Channels and Variation in Motivations for Channel Selection: Cross-sectional Survey
Source: J Med Internet Res. 2021 Mar 9;23(3):e24945. doi: 10.2196/24945 (PMC7988389; doi:10.2196/24945)
Supplement: Multimedia Appendix 1 [file jmir_v23i3e24945_app1.docx]

**Appendix 1: Measurement Model Building Process**

Three measurement models were constructed and compared (see Table below).

| **Table. Model Fit Statistics** | | | | | | | |
| --- | --- | --- | --- | --- | --- | --- | --- |
|  | *Model* | *df* | $\chi^{2}$ | $\chi^{2}$*/df* | *RMSEA* | *CFI* | *SRMR* |
| **Measurement Model** | 1. Baseline (2-factor) | 303 | 1149.05 | 3.79 | .072 | .86 | .059 |
|  | 2. Revised (3-factor) | 296 | 904.82 | 3.06 | .062 | .90 | .052 |
|  | 3. Revised + Corr. Error | 290 | 637.95 | 2.20 | .047 | .94 | .049 |
| **Structural Model** | 1. Baseline conceptual | 343 | 922.97 | 2.69 | .056 | .91 | .081 |
|  | 2. Revised | 351 | 931.68 | 2.65 | .055 | .91 | .083 |
| *Recommended cutoff points for model fit indices (Byrne, 2001; Hu & Bentler, 1999; Kline, 1998): SRMR <.08; RMSEA < .08; CFI > .90 (ideally CFI$\geq$.95); $\chi^{2}$*/df* <3. Hu and Bentler (1999) suggest a two-index presentation strategy, recommending RMSEA of .06 or lower and a SRMR of .09 or lower.  ** 2 factor: active seeking and scanning channels  *** 3 factor: searching, browsing and scanning channels | | | | | | | |

**Measurement Model 1:** The 13 channel use variables were broken down to two latent variables (active use of channels vs. non-active use of channels). The commonly used model fit indices such as χ^2^/df and CFI for the 2-factor baseline measurement model in the table above suggest unsatisfactory model fit.

**Measurement Model 2:** The 13 channel use variables were classified into three latent variables: use of browsing, searching and scanning channels. According to the table above, the model fit indices for the 3-factor measurement model shows improvement over the 2-factor model. However, χ^2^/df is still higher than 3.

**Measurement Model 3:** The 13 channel use variables were still classified into three latent variables: use of browsing, searching and scanning channels. Additionally, the error terms of six pairs of channel use variables were correlated, which follows the following logic. An individual user who actively seeks health information from a particular channel is more likely to incidentally encounter health information from that particular channel while using it for other tasks unrelated to health. In this study, some of the online health information channels are based on the same genre of media platforms. For instance, active use of WeChat (reading content published by WeChat Accounts specialized in health topics) and nonactive (passive) use of WeChat (incidental exposure to health-related updates or shared messages in WeChat Moments) are based on the mobile application WeChat. A habitual WeChat user is likely to use WeChat to acquire health information actively and passively; statistically speaking, they are moderately or highly correlated. The six pairs can be found in the first table in Appendix 1. The table above shows that the model fit of measurement model 3 with correlated errors outperforms the 3-factor measurement model. Thus, the structural model was built on basis of measurement model 3.
