# Supplementary material for: Classification of the Use of Online Health Information Channels and Variation in Motivations for Channel Selection: Cross-sectional Survey
Source: J Med Internet Res. 2021 Mar 9;23(3):e24945. doi: 10.2196/24945 (PMC7988389; doi:10.2196/24945)
Supplement: Multimedia Appendix 2 [file jmir_v23i3e24945_app2.docx]

**Appendix 2: Tables of Bivariate Correlations**

| **Table. Zero-order correlations among use of online health information channels (*N*=542)** | | | | | | | | | | | | | | | |
| --- | --- | --- | --- | --- | --- | --- | --- | --- | --- | --- | --- | --- | --- | --- | --- |
|  |  | *b.* | | *c.* | *d.* | *e.* | *f.* | *g.* | *h.* | *i.* | *j.* | *k.* | *l.* | | *m.* |
| a. | Health sections of web portals | **.71** | | .50 | .43 | .58 | .57 | .26 | .39 | .37 | **.60** | .41 | .23 | | .50 |
| b. | Professional health sites |  | | .40 | .38 | .60 | .57 | .31 | .36 | .38 | **.49** | .39 | .21 | | .52 |
| c. | Microblogs (i.e. health microblogs) | | | | .57 | .49 | .50 | .23 | .37 | .37 | .46 | **.64** | .28 | .45 | |
| d. | WeChat official accounts | |  | |  | .45 | .47 | .33 | .36 | .40 | .42 | .45 | **.41** | .45 | |
| e. | Mobile health apps |  | |  |  |  | .55 | .22 | .37 | .35 | .43 | .43 | .21 | | .54 |
| f. | Online forums (i.e. health forums) | | | | |  |  | .30 | .45 | .40 | .51 | .44 | .27 | **.63** | |
| g. | Search engines |  | |  |  |  |  |  | .51 | .53 | .27 | .26 | .30 | | .30 |
| h. | Encyclopedia sites |  | |  |  |  |  |  |  | .61 | .43 | .36 | .34 | | .44 |
| i. | Q&A sites |  | |  |  |  |  |  |  |  | .37 | .36 | .33 | | .46 |
| j. | Web portals |  | |  |  |  |  |  |  |  |  | .52 | .43 | | .53 |
| k. | Microblogs |  | |  |  |  |  |  |  |  |  |  | .36 | | .51 |
| l. | WeChat Moments |  | |  |  |  |  |  |  |  |  |  |  | | .36 |
| m. | Online forums |  | |  |  |  |  |  |  |  |  |  |  | |  |
| *All the coefficients are statistically significant at .05 level.  **The coefficients in bold suggest that the error terms of these pairs of channel use variables are correlated with each other in building measurement models. | | | | | | | | | | | | | | | |

| **Table. Zero-order correlations between channel use and predictors (*N*=542)** | | | | | | | |
| --- | --- | --- | --- | --- | --- | --- | --- |
|  | PR | ISN | NA | RCB | PIGC | CK | IST |
| Health sections of web portals | -.04 | **.30** | **.14** | **.42** | **-.20** | **.18** | -.01 |
| Pro. health sites | -.02 | **.32** | **.13** | **.46** | **-.22** | **.16** | .00 |
| Microblogs (i.e. health microblogs) | -.02 | **.25** | **.26** | **.40** | **-.22** | **.11** | -.02 |
| WeChat official accounts | -.04 | **.31** | **.12** | **.42** | **-.13** | **.15** | **.09** |
| Mobile health apps | -.02 | **.33** | **.16** | **.41** | **-.25** | **.14** | -.02 |
| Online forums (i.e. health forums) | -.01 | **.33** | **.19** | **.38** | **-.27** | **.17** | -.02 |
| Search engines | -.02 | **.19** | .06 | **.33** | .03 | **.09** | **.15** |
| Encyclopedia sites | .08 | **.27** | **.12** | **.37** | **-.09** | **.12** | **.15** |
| Q&A sites | -.04 | **.26** | **.15** | **.33** | **-.12** | **.09** | **.09** |
| Web portals | -.03 | **.29** | .07 | **.40** | **-.09** | **.19** | .02 |
| Microblogs | .03 | **.30** | **.09** | **.41** | -.07 | **.11** | .03 |
| WeChat Moments | -.02 | **.25** | .08 | **.32** | .01 | **.17** | **.20** |
| Online forums | .02 | **.30** | **.16** | **.43** | **-.24** | **.11** | .02 |
| *PR=perceived risks; ISN=information subjective norms; NA=negative affect; RCB= Relevant channel beliefs; PIGC=perceived information gathering capacities; CK= current knowledge; IST= information sufficiency threshold  **Coefficients in bold are statistically significant at .05 level. | | | | | | | |
